# Research on stress curve clustering algorithm of Fiber Bragg grating sensor

**DOI:** 10.1038/s41598-023-39058-w

**Published:** 2023-07-21

**Authors:** Yisen Lin, Ye Wang, Huichen Qu, Yiwen Xiong

**Affiliations:** School of Computer Science and Engineering, Guilin University of Aerospace Technology, Guilin, 541004 China

**Keywords:** Electrical and electronic engineering, Computer science

## Abstract

The global stress distribution and state parameter analysis of the building's main structure is an urgent problem to be solved in the online state assessment technology of building structure health. In this paper, a stress curve clustering algorithm of fiber Bragg grating stress sensor based on density clustering algorithm is proposed. To solve the problem of large dimension and sparse sample space of sensor stress curve, the distance between samples is measured based on improved cosine similarity. Aiming at the problem of low efficiency and poor effect of traditional clustering algorithm, density clustering algorithm based on mutual nearest neighbor is used to cluster. Finally, the classification of the daily stress load characteristics of the sensor is realized, which provides a basis for constructing the mathematical analysis model of building health. The experimental results show that the stress curve clustering method proposed in this paper is better than the latest clustering algorithms such as HDBSCAN, CBKM, K-mean++,FINCH and NPIR, and is suitable for the feature classification of stress curves of fiber Bragg grating sensors.

## Introduction

Fiber Bragg sensor (FBG) was widely used in national defense, railway, chemical industry, environment, nuclear power, bridge and tunnel monitoring and other fields due to its characteristics of low transmission loss, corrosion resistance, good insulation and electromagnetic interference resistance^1, 2^. Building structure safety monitoring system can obtain large and accurate stress and temperature data of building structure through fiber grating stress sensor. Because the geological and environmental changes are a slow evolutionary process, it is not feasible to judge the safety of building structures from the monitoring data at a certain point in time. Only through the accumulation of reliable data collection and the correct mathematical model analysis can we make the safe prediction with high reliability. By using the existing data mining and analysis technology, the daily stress load data of building stress sensor is analyzed and features are extracted by clustering analysis algorithm, and a mathematical analysis model can be constructed to evaluate and predict the overall health status of buildings. This is of great significance to the construction safety monitoring scenario.

The FBG sensor is mainly formed by using the ultraviolet exposure technology to induce periodic changes in the optical refractive index in the fiber core according to the photosensitive characteristics of the fiber material^3–5^. The periodic change of the optical refractive index distribution in the FBG leads to the reflection of light of a certain wavelength, which is equivalent to forming a narrow-band filter in the optical fiber, thereby forming the reflection spectrum of the FBG. FBGs only reflect light at the Bragg wavelength, and the reflected Bragg wavelength is proportional to the temperature and strain value.

**Figure 1:**
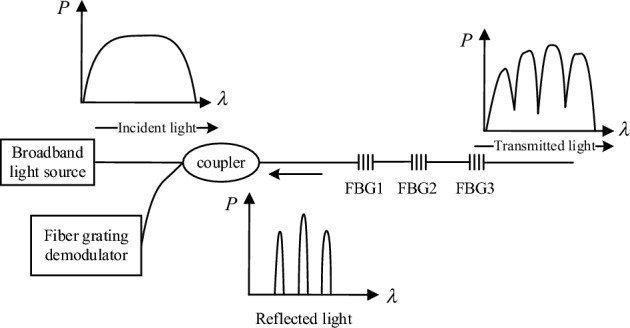
Schematic diagram of working principle of FBG demodulation.

The working principle of the sensor is shown in Fig. 1. When the light source enters the FBG through the optical fiber for coupling, the FBG will selectively reflect back light of a specific wavelength, and the reflected Bragg wavelength ${\lambda }_{B}$ can be expressed as ^3^

1 $${\lambda }_{B}=2{n}_{eff}\Lambda$$

In Eq. (1), ${\lambda }_{B}$ is the center wavelength of the reflected light, ${n}_{eff}$ is the effective refractive index of the fiber, and $\Lambda$ is the period of the fiber grating. It can be found from this that ${\lambda }_{B}$ changes with the changes of ${n}_{eff}$ and $\Lambda$, and the effective refractive index of the fiber and the period of the fiber grating are very easily affected by the ambient temperature and the stress of the fiber grating sensor. The offset $\Delta {\lambda }_{B}$ of the center wavelength of ${\lambda }_{B}$ can be expressed as

2 $$\Delta {\lambda }_{B}={\lambda }_{B}[\left(1-{P}_{e}\right)\varepsilon +(\alpha +\xi )\Delta T]$$

In Eq. (2), $\varepsilon$ is the axial strain of the fiber grating, $\alpha$ is the thermal expansion coefficient of the fiber, $\xi$ is the thermal optical coefficient of the fiber, $\Delta T$ is the temperature change. ${P}_{e}$ is called the effective photoelastic coefficient, and can be expressed as

3 $${P}_{e}=\frac{{n}_{eff}^{2}}{2}[{P}_{12}+\upsilon ({P}_{11}+{P}_{12})]$$

In Eq. (3), $\upsilon$ is the Poisson's ratio. When using a fiber with pure silica core and boron dioxide doped cladding, ${P}_{11}=0.121$, ${P}_{12}=0.27$, $\upsilon =0.17$. When the effective refractive index of the fiber is ${n}_{eff}=1.46$, the effective photoelastic coefficient ${P}_{e}=0.22$. When $\Delta {\lambda }_{B}$ is measured by a fiber grating demodulator, its axial stress change $\varepsilon$ can be expressed as^3^

4 $$\varepsilon =1/(1-{P}_{e} )\cdot (\Delta {\lambda }_{B})/{\lambda }_{B}$$

## Related work

### Stress data processing method of FBG sensor

There are many methods for structural health monitoring in different scenarios. Kahandawa et al.^3^ proposed a fixed filter decoding system and an integrated artificial neural network algorithm for extracting strain from embedded FBG sensor. On the basis of Kalman filter, Song et al.^4^ taked the strain value measured by FBG sensor as the observed signal. Through the gain matrix, new information sequence and covariance matrix generated by Kalman filter, the least square algorithm is used to estimate the load size in real time. However, this algorithm can only calculate the real-time local load of the building, and cannot give the current overall health status of the building based on the past sensor data. Zhang et al.^5^ proposed a model reconstruction predicting algorithm based on PSO-SVR to achieve the self-repairing of the FBG sensor network in SHM system. Through this algorithm, the reliability and survivability of the FBG-based SHM system is enhanced if partial FBG sensors are invalid. Stotaw Talbachew Hayle et al.^6^ used deep learning technique to accurately identify the Bragg wavelength of FBGs in the condition of the partially or fully overlapped spectra to improve the reliability and detection accuracy of the sensor system even the number of overlaps FBGs spectra increases. Jiang et al.^7^ developed an FBG sensing-based structural health monitoring system for Chinese ancient Chuan-dou-type timber buildings that aims at monitoring structural deformation. Methods proposed by Zhang et al.^8^ can accurately identify structural macro strain modal shapes and are much more robust than traditional modal parameter based indexes for structural damage detection. Sierra-Pérez et al.^9^ presented a damage detection methodology based on optimal baseline selection by means of clustering techniques which includes the use of hierarchical nonlinear PCA as a nonlinear modeling technique. Luckey et al.^10^ proposed conceptual explainable artificial intelligence framework that provide a basis for improving ML acceptance and transparency and therefore increase trust in ML algorithms implemented in SHM applications.

Most of the above methods used FBG sensors to monitor loads in different scenarios in real time, without assessing or predicting the overall health status of buildings from a global perspective. So this paper puts forward a scheme to solve this problem.

### Clustering algorithms

Clustering algorithm is a commonly used unsupervised learning algorithm and can be used to find hidden patterns in datasets. The purpose of clustering is to divide the samples in a dataset into several disjoint subsets. The data of fiber grating building stress sensor is usually collected at a certain time interval. The stress data collected from the same sensing point exists in the form of curves. In this paper, the daily load stress curve of stress sensor is taken as a sample, and the clustering algorithm is used to analyze it, and the mathematical model of building health is built. Traditional clustering algorithms have low clustering efficiency and poor clustering effect^11–13^. For example, the clustering effect of K-means algorithm^14^ depends heavily on the selection of cluster centers. The clustering results often fall into local optimum due to inappropriate initial cluster centers or the influence of noise and boundary points, and cannot adapt to non-convex shape data, and the K-means algorithm also needs to specify the number of clusters^15, 16^. CBKM^17^ is an improved algorithm of KMeans algorithm. It improves clustering by using better initialization and iteration. However, CBKM cannot correctly classify non-spherical data. The traditional density clustering algorithm DBSCAN^18^ needs to specify the scanning radius (eps) and the minimum number of included points (minPts) for different data, which requires researchers to test one by one, and it is difficult to adjust parameters^19–22^, resulting in low clustering efficiency. HDBSCAN^23^ is a hierarchical algorithm. It provides a clustering hierarchy, constructs a clustering tree on this basis, and then extracts clustering from the optimal local cut through the clustering tree. A state-of-the-art algorithm in hierarchical algorithm is the FINCH algorithm^24^. In this algorithm, an adjacency matrix is defined according to the clustering equation to join the points in the point data set. In proximity-based algorithms, FastDP algorithm^25^ uses a fast and generic construction of approximate k-nearest neighbor graph to improve the quadratic time complexity of the DPC algorithm^26^. Another latest algorithm based on DPC is NPIR algorithm^27^. The algorithm is based on nearest neighbor search to deal with clustering problems. This algorithm requires three parameters: the number of clusters, index ratio and iteration times.

These algorithms can not reflect the potential law and change characteristics of fiber stress data well, and can not provide good support for building health mathematical analysis model. The density clustering algorithm based on inverse nearest neighbor proposed in this paper can solve these problems existing in the traditional clustering algorithm in the FBG building stress data scene.

## Proposed method

### Density clustering algorithm based on mutual nearest neighbor

This subsection mainly describes the specific process of mutual nearest neighbor-based density estimation clustering. Given the number of neighbors *k*, *k* is a positive integer, and the distance between $x$ and $y$ is $d\left(x,y\right)$. Assuming that the sample set is S, the set of k nearest neighbors of a sample point x in the set, that is, the set of k sample points closest to the sample point $x$ is expressed as:

5 $${NN}_{k}\left(x\right)=\{{s}_{1},\dots {s}_{k}\in S|d\left({s}_{i},x\right)\le d(z,x)\forall z\in S\backslash \{{s}_{1},\dots {s}_{k}\}\}$$

The k-inverse neighbor set of sample point $x$ is expressed as:

6 $${RNN}_{k}\left(x\right)=\{z\in S|x\in {NN}_{k}\left(z\right)\}$$

The k-mutual neighbor set of sample point $x$ is expressed as:

7 $${MNN}_{k}\left(x\right)={NN}_{k}\left(x\right){\cap RNN}_{k}\left(x\right)$$

Algorithm 1 describes the overall clustering algorithm improved from^28^, followed by a detailed discussion of its time complexity


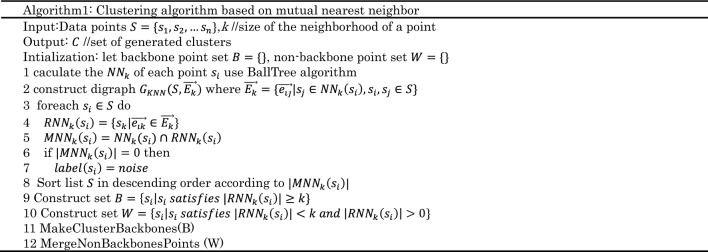



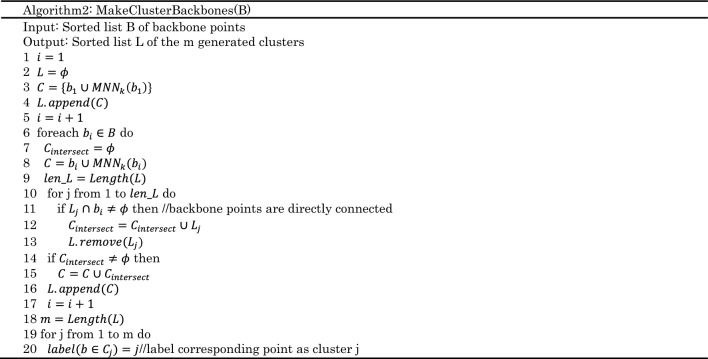



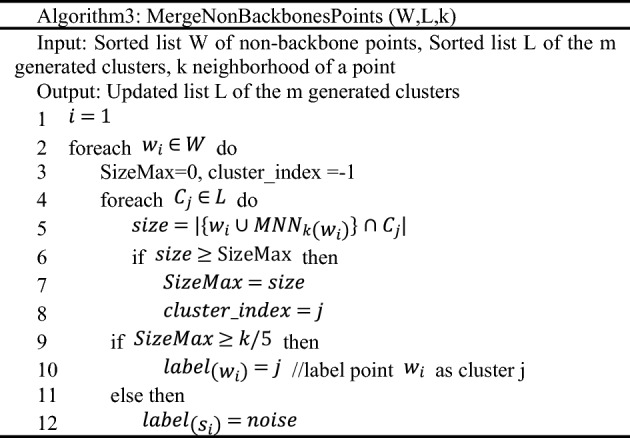


Algorithm 1 consists of the following two stages:

(1) The first stage:

$P$ are sorted based on $|{RNN}_{k}\left(x\right)|$ in a descending order.

Points in dataset which have the property of $|M{NN}_{k}\left({p}_{i}\right)|=0$ are labeled as noise points.

MakeClusterBackbones(Algorithm2): After removing the noise points with $|M{NN}_{k}\left({p}_{i}\right)|=0$, the remaining points are divided into backbone points and non-backbone points. Backbone points are used to construct the skeleton of a cluster and non-backbone points are processed in the second stage. When two backbone points are mutual neighbors ,they are connected to form the skeleton of the same cluster.

(2) Merge Non Backbones Points (Algorithm3): For each non-backbone point ${w}_{i}$, find out the $M{NN}_{k}({w}_{i})$ of that contains the most backbone points belongs to certain cluster. If there are some backbone points belongs to a certain cluster in $M{NN}_{k}({w}_{i})$ and the number of that is greater than or equal to $k/5$, it is determined that it belongs to the certain cluster. Otherwise, ${w}_{i}$ is marked as a noise.

### Time complexity

Suppose that the number of points in the dataset is *N*, the number of nearest neighbors is *k*, the dimension of the data is *D*, and the number of clusters constructed is *C*. The time complexity for constructing the distance matrix is $O\left(NlogN\right)$^28^ and^29^. In algorithm 1, it takes K iterations to calculate both ${NN}_{k}\left({p}_{i}\right)$ and ${RNN}_{k}\left({p}_{i}\right)$ of each point, so the time complexity of this part is $O\left(Nk\right)$^30^. The time complexity analysis of the remaining part of algorithm 1 is as follows:

1. Line8, according to ${|MNN}_{k}\left({p}_{i}\right)|$ descending to the time complexity is $O\left(NlogN\right)$ to sort the list of $P$.
2. Line11, the complexity of MakeClusterBackbones(Algorithm2) is $O\left(\left|S\right|*\left|U\right|*logk\right)$,where $|U|$ is an upper limit of the number of generated clusters^31^. Consider the worst case, $O\left(\left|U\right|\right)=O(|S|)$ and $O\left(\left|S\right|\right)=N$. Finally, the overall complexity is $O\left({N}^{2}logk\right)$.
3. Line12, the complexity of MergeNonBackbonesPoints (Algorithm3) is $O\left(\left|W\right|*\left|U\right|*k\right)$^31^. Based on the same analysis, the complexity is $O\left({N}^{2}k\right)$.

In summary, the overall time complexity for Density clustering algorithm is $O\left({N}^{2}k\right)$.

### Pattern recognition algorithm for stress curves of fiber bragg grating sensors

**Figure 2:**
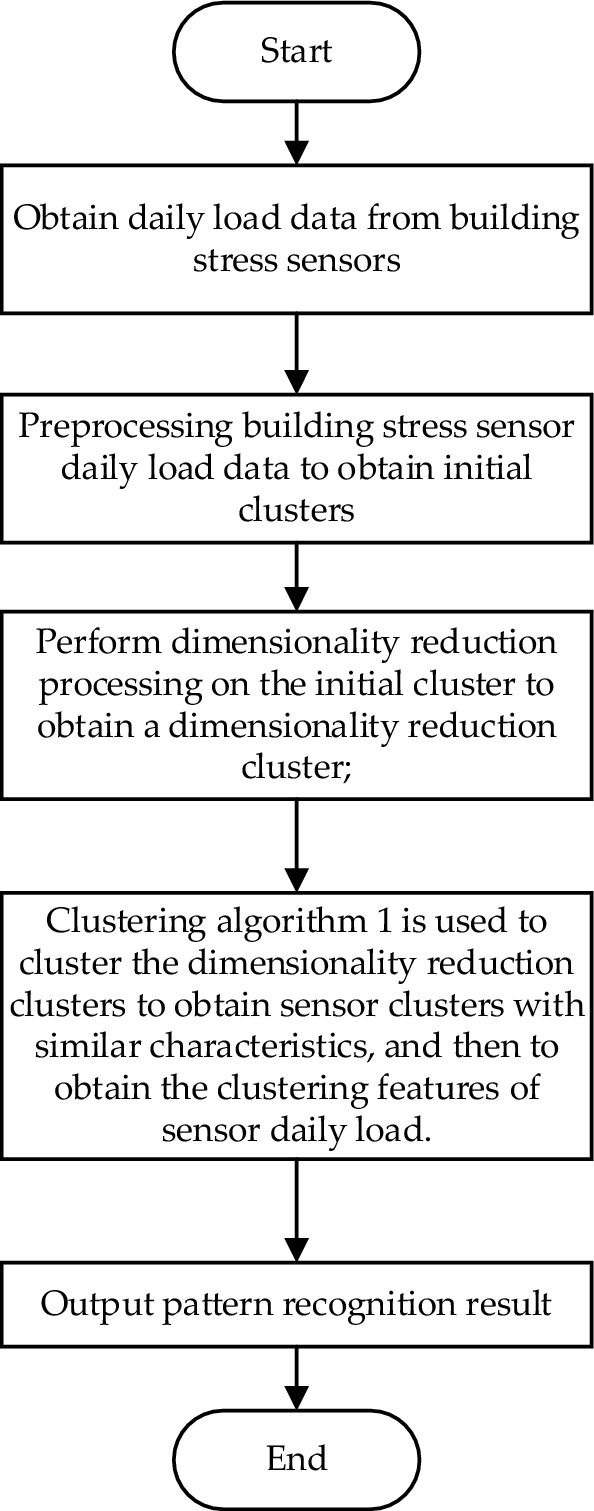
Pattern recognition algorithm flow of the stress curve.

Figure 2 shows the pattern recognition algorithm flow of the stress curve of the fiber grating building stress sensor based on the mutual neighbor clustering algorithm, in which the clustering adopts the algorithm described in 3.1.

Firstly, the daily load data set of the building stress sensor is obtained. The data set contains P samples, and each sample has a data set matrix of Q time point attributes. The building health monitoring system collects sample data through fiber grating sensors installed in the building, and each sample is the stress daily load of a sensor.

Secondly, preprocess the acquired daily load data of the building stress sensor to obtain an initial cluster, wherein the preprocessing progress includes missing value processing, data standardization, data regularization and data dimensionality reduction on the daily load data of the building stress sensor. After this step, the initial cluster obtained is a dataset matrix of p samples with q attributes per sample.

Specifically, the missing value processing is to delete the samples with few valid values, and complete the missing values of the samples with many valid values. Of course, when deleting attributes with few valid values, redundant attributes can be deleted together.

In the process of deleting samples, if n samples are deleted, p samples remain, where *p* = *P* − n. In addition, there are various ways to supplement the missing values. In this application, the average of the existing validity is taken as the filling value of the missing value. Those skilled in the art can choose other supplementary methods, which will not affect the subsequent analysis process.

Data normalization is to linearize the stress part data in the original data into the range of [− 1, 1]. The calculation formula of the maximum-minimum normalization is:

8 $${X}_{norm}=1/(1-{e}^{-X})$$

This formula realizes the proportional scaling of the original data, where ${X}_{norm}$ is the normalized data, and $X$ is the original data.

Data regularization is to subtract each attribute from the corresponding mean of the attribute, and then divide by the corresponding variance of the attribute. After standardization and regularization, the data of each attribute are clustered around 0 and the variance is 1, that is, the obtained sample data has zero mean and unit variance.

Data dimensionality reduction is to use PCA (Principal Component Analysis), to reduce the dimensionality of the data set, and obtain the processed dimensionality reduction cluster S. In the process of dimensionality reduction, if the number of dimensionality reduction is *q*, then the dimensionality reduction cluster *S* after dimensionality reduction is a dataset matrix with p samples and each sample has q attributes.

A clustering algorithm is used to classify the data in the data set *S*, so as to identify sensor groups with similar characteristics. Using the density estimation clustering algorithm based on mutual neighbors, the p samples in the dataset are divided into M classes, where M is a positive integer. Since the dimension of the sample data after dimensionality reduction is still large, in order to avoid the problem of sparse sample space, the distance between the samples is calculated by the improved cosine similarity:

9 $$d\left(x,y\right)=\frac{xy}{|x||y|}=\frac{\sum_{i=1}^{q}{x}_{i}{y}_{i}}{\sqrt{\sum_{i=1}^{q}{x}_{i}^{2}}\sqrt{\sum_{i=1}^{q}{y}_{i}^{2}}}$$

The Calinski-Harabasz (CH) value is used to evaluate the clustering effect, and the result with the largest CH value is selected, and finally M classes of sensor clusters are obtained. The specific calculation process of Calinski-Harabasz (CH) is as follows:

10 $$CH=\frac{trB(h)/(h-1)}{trW(h)/(m-h)}$$

In the above formula, m represents the number of clusters, h represents the current class, trB(h) represents the trace of the inter-class dispersion matrix, and trW(h) represents the trace of the intra-class dispersion matrix. The larger the CH value, the closer the samples within the class, the more dispersed the samples between different classes, that is, the better clustering results.

## Experimental results

In order to verify the feasibility and superiority of the proposed algorithm, K-Means++, CBKM, HDBSCAN, FINCH, NPIR and the proposed algorithm are used to cluster the data sets with known classification labels and the actual collected data of fiber grating sensor, and then the clustering effect is analyzed.

### Analysis of clustering results of known classification labeled data sets

**Figure 3:**
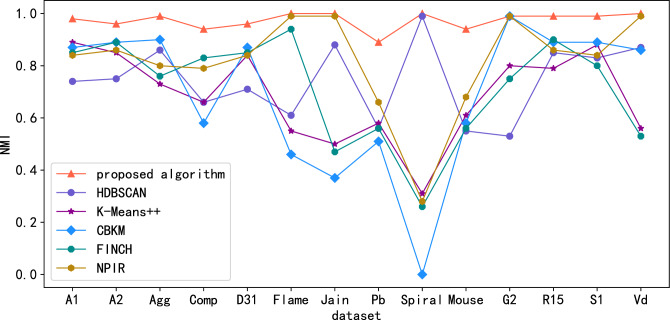
Comparison of NMI scores in standard data sets.

**Figure 4:**
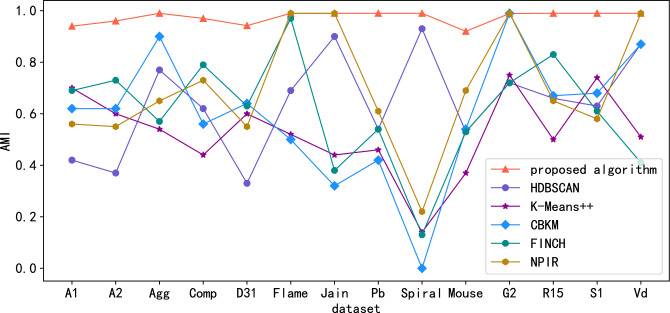
Comparison of AMI scores in standard data sets.

**Figure 5:**
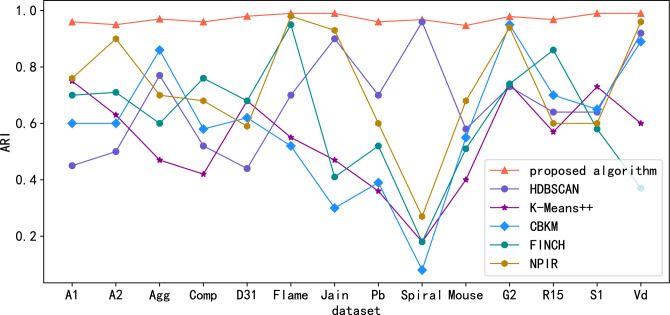
Comparison of ARI scores in standard data sets.

**Figure 6:**
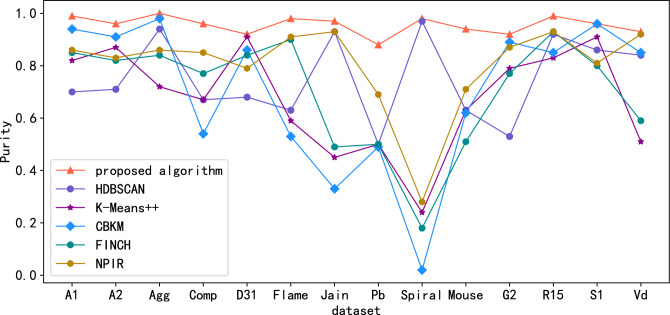
Comparison of purity scores in standard data sets.

**Figure 7:**
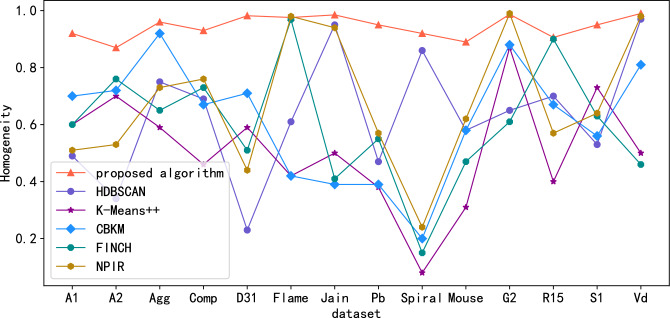
Comparison of homogeneity scores in standard data sets.

**Figure 8:**
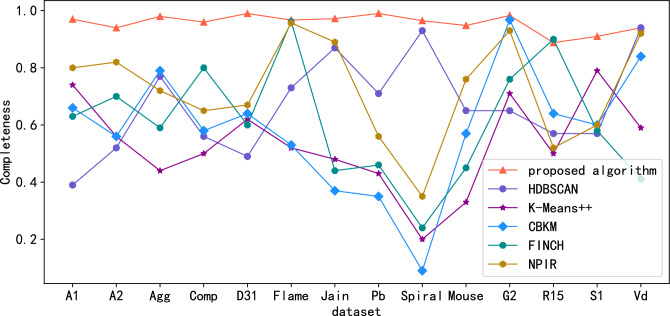
Comparison of completeness scores in standard data sets.

Public standard datasets with known classification labels (witch includes A1, A2, Aggregation(Agg), Compound(Comp), D31, Flame, Jain, Pathbased(Pb), Spiral, Mouse, G2-2-10(G2), R15, S1, Vary density(Vd)) are used to evaluate algorithm effectiveness. Since the Calinski-Harabasz (CH) metric is suitable for situations where the actual classification label is unknown. For datasets with known classification labels, the clustering effect index can be measured by Normalized Mutual Information (NMI) , Adjusted Mutual Information (AMI), Adjusted Rand Index(ARI), Purity, Homogeneity and Completeness scores. Figures. 3, 4, 5, 6, 7 and 8 shows the results of clustering experiments on datasets with classification labels.

### Analysis of clustering results of actual collected data

**Figure 9:**
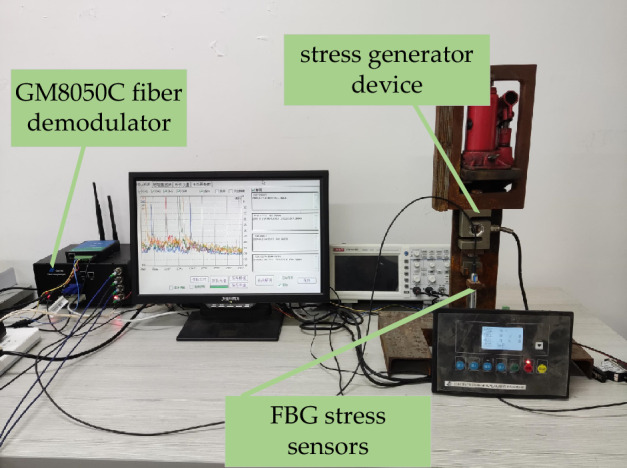
Experimental platform.

The experimental device platform used in this paper to collect fiber grating sensor data consists of fiber demodulator, FBG sensor, and stress generator. The experimental platform is shown in Fig. 9. The FBG sensor is placed in the stress generator device which can apply different types of force shocks to the FBG sensor through programming. The center wavelength of the FBG is demodulated through the GM8050C fiber demodulator to calculate the stress value. The GM8050C fiber demodulator has 4 channels, each of which can be connected to 16 FBG stress sensors in series.

In this paper, the stress curves of 4 FBG sensors are collected as data samples through the experimental device platform. The collection time interval is 2 min and the collection period is 24 h. The daily load data of the stress sensor consists of 720 stress data. Each stress curve sample is collected continuously for 3 months. The proposed algorithm, K-Means++, HDBSCAN,CBKM,FINCH and NPIR algorithms were used to compare the clustering effect of the daily load data of S1, S2, S3, S4 sensors and the mixed data of the four sensors respectively. The optimal results of the Calinski-Harabasz(CH) index obtained by the experimental test are shown in Table 1.

**Table 1:** The CH results of the proposed algorithm and other five algorithms.

Sensor data	S1	S2	S3	S4	S1 + S2 + S3 + S4
Proposed algorithm	1271.57	1068.29	1156.61	978.39	1006.26
HDBSCAN	931.73	789.24	804.19	902.26	857.48
K-Means++	765.28	727.53	786.04	704.24	752.86
CBKM	812.67	794.33	826.74	873.36	849.73
FINCH	1147.85	984.04	953.68	949.75	979.45
NPIR	998.52	1021.71	1045.64	930.83	985.72
Rank	1	1	1	1	1

### Result and discussion

Experimental results show that proposed algorithm greatly outperforms other algorithms on many datasets such as A1, A2, Aggregation, Compound, D31, Pathbased, Mouse, R15, S1. The performance of NPIR, HDBSCAN and CBKM on Frame, Jain, Spiral, G2-2-10, Vary density datasets is almost equal to that of the proposed algorithms. This is due to the framework adopted by the proposed algorithm in the clustering process can distinguish real clusters in noisy data, even if they are connected to each other or even overlap, provided that they have distinguishable densities. The clustering effect of HDBSCAN deteriorates at different densities between clusters because of the fixed neighborhood radius. Centroid based algorithms like K-Means++and CBKM fail when the centroid of a cluster is closer to the data points belongs to other cluster than to that of its own cluster. FINCH will output incorrect clustering results due to inappropriate location of the merged cluster center during hierarchical merging. NPIR perform poorly when the data is noisy or the cluster density is different and slightly overlapped. The process of calculating the ${MNN}_{k}$ of each point in the proposed algorithm is actually constructing the structure of mutual neighbor graph, and then completing the clustering by dividing the subgraphs. The structure of the mutual nearest neighbor graph is determined only by the parameter K, and there are no hyperparameters related to the distance between points. Therefore, the algorithm still performs well when the cluster density is different or there are connections between clusters.

The last row of the Table1 shows the ranking of the proposed algorithm in the performance of all six algorithms based on Calinski-Harabasz (CH) index. The results show that the CH value of the algorithm in this paper are both greater than that of other five algorithms, which proves that the clustering effect of the algorithm in this paper is suitable for the actual sensor daily load curve. In general, it can be seen that the clustering effect of the proposed algorithm in this paper is better than that other five algorithms, and the data of the fiber grating stress sensor can be clustered with higher accuracy. However, the proposed algorithm still has problem: (1)the *k* value for the nearest neighbor in the the algorithm still cannot be determined adaptively. (2)The time complexity of the algorithm is still unacceptably high when large samples need to be processed.

## Conclusions

Aiming at the clustering problem of the stress curve of the fiber grating stress sensor, this paper obtains the initial cluster by preprocessing the stress data of the fiber grating building stress sensor. The initial cluster is dimensionally reduced to obtain a dimensionality-reduced cluster. The initial clusters are clustered by the clustering algorithm based on mutual neighbors, and the clustering effectiveness index is used to evaluate the clustering results, and finally sensor groups with similar characteristics are obtained. The cosine similarity is used to calculate the sample distance based on the mutual neighbor clustering algorithm, which solves the problem of sparse sample space caused by the large dimension of the sample data. The experimental results show that compared with the other five clustering algorithms, the proposed algorithm has a better pattern recognition effect.

## Data Availability

The datasets with known classification labels (witch includes A1, A2, Aggregation, Compound, D31, Flame, Jain, Pathbased, Spiral, Mouse, G2-2–10, R15, S1, Vary density) analysed during the current study are available in http://cs.joensuu.fi/sipu/datasets/ and https://elki-project.github.io/datasets/.The data of FBG sensors analysed during the current study are not publicly available due to the Lab's Policy or Non-Disclosure Agreement but are available from the first author(Yisen Lin, Email:linyisen@guat.edu.cn) on reasonable request.
