# Author Correction: Research on stress curve clustering algorithm of Fiber Bragg grating sensor

**DOI:** 10.1038/s41598-023-42289-6

**Published:** 2023-09-20

**Authors:** Yisen Lin, Ye Wang, Huichen Qu, Yiwen Xiong

**Affiliations:** School of Computer Science and Engineering, Guilin University of Aerospace Technology, Guilin, 541004 China

Correction to: *Scientific Reports* 10.1038/s41598-023-39058-w, published online 21 July 2023

The original Article contained errors.

Algorithms 1 and 2 were incomplete. The original Algorithm 1 appears below:
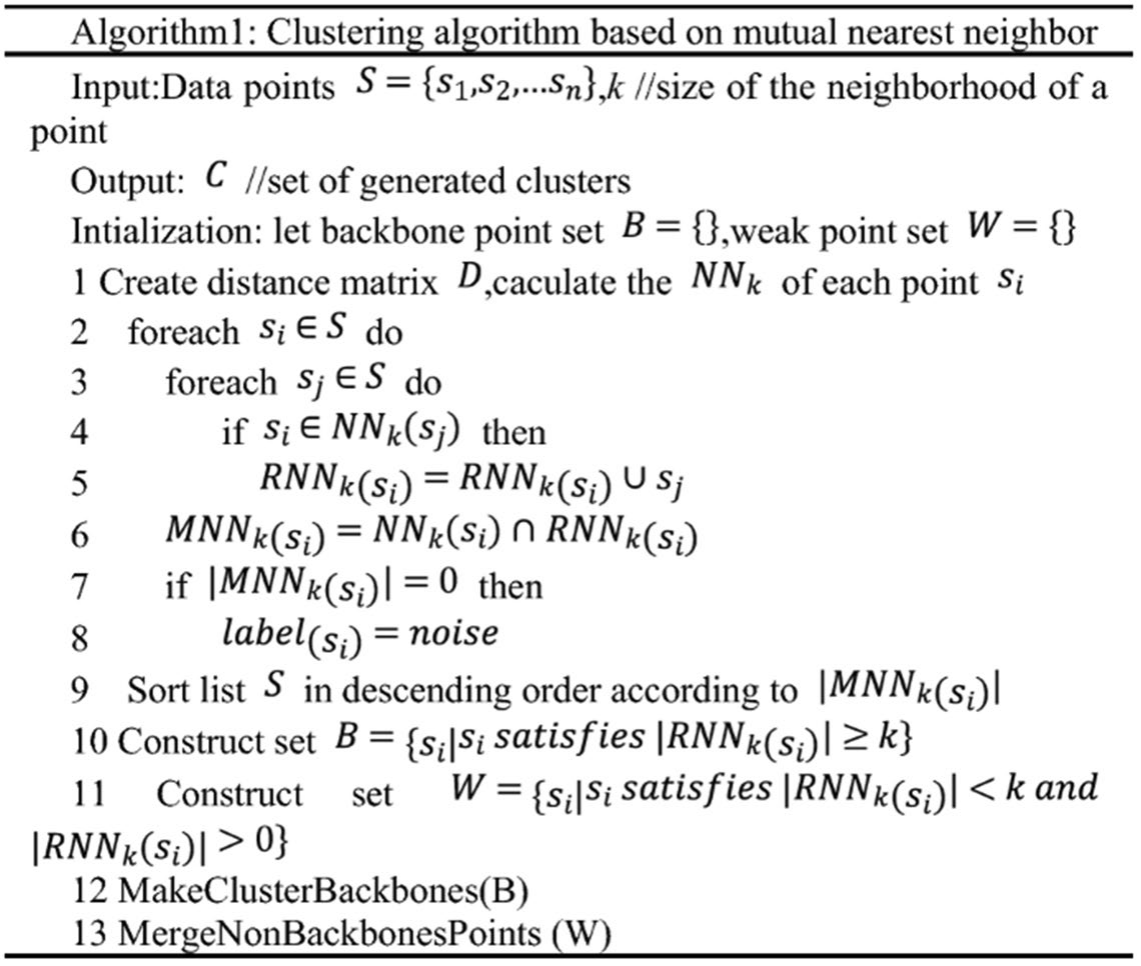


The original Algorithm 2 appears below:
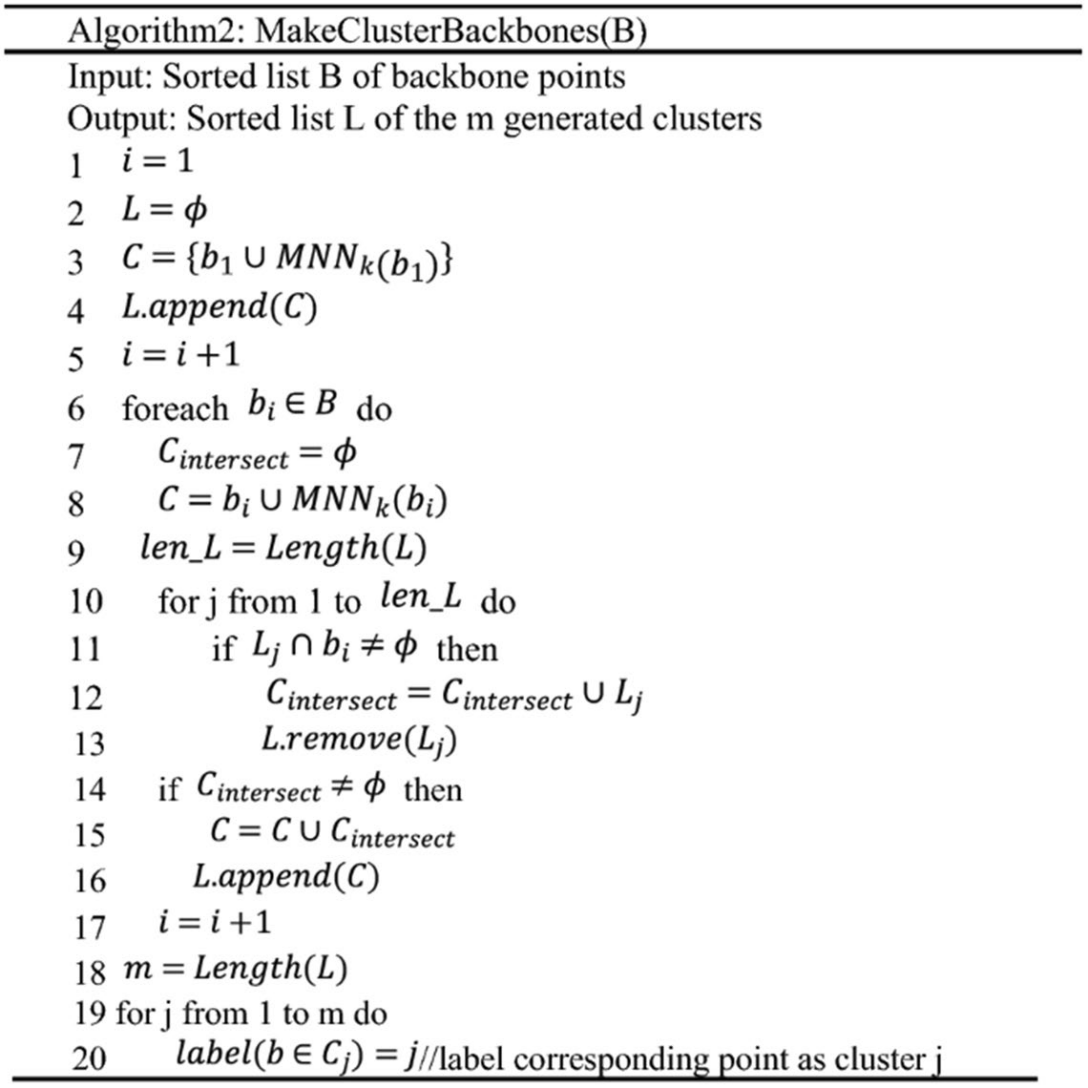


Also in the Reference list. Reference [30] should have been cited earlier and therefore becomes reference [28].

In the section “Proposed method” under the subheading “Density clustering algorithm based on mutual nearest neighbour” after equation (7) the following sentence was omitted:

Algorithm 1 describes the overall clustering algorithm improved from [28], followed by a detailed discussion of its time complexity.

In the section “Proposed method” under the subheading “Time complexity”, the sentence.

(1) Line9, according to ${|MNN}_{k}\left({p}_{i}\right)|$ descending to the time complexity is $O\left(NlogN\right)$ to sort the list of $P$.

Now reads:

(1) Line8, according to ${|MNN}_{k}\left({p}_{i}\right)|$ descending to the time complexity is $O\left(NlogN\right)$ to sort the list of $P$.

The sentence:

(2) Line12, the complexity of MakeClusterBackbones(Algorithm2) is $O\left(\left|S\right|*\left|U\right|*logk\right)$, where $|U|$ is an upper limit of the number of generated clusters [31].

Now reads:

(2) Line11, the complexity of MakeClusterBackbones(Algorithm2) is $O\left(\left|S\right|*\left|U\right|*logk\right)$, where $|U|$ is an upper limit of the number of generated clusters [31].

And the sentence:

(3) Line13, the complexity of MergePoints (Algorithm3) is $O\left(\left|W\right|*\left|U\right|*k\right)$ [31].

Now reads:

(3) Line12, the complexity of MergeNonBackbonesPoints (Algorithm3) is $O\left(\left|W\right|*\left|U\right|*k\right)$ [31].

The original references [28] and [29] have been renumbered.

The original Article has been corrected.

